# PROMETHEUS: Long-Term Exacerbation and Mortality Benefits of Implementing Single-Inhaler Triple Therapy in the US COPD Population

**DOI:** 10.36469/001c.55635

**Published:** 2023-01-24

**Authors:** Gerard Criner, Fernando Martinez, Hitesh Gandhi, Norbert Feigler, Bruce Pyenson, Matthew Emery, Umang Gupta, Muthiah Vaduganathan

**Affiliations:** 1 Temple University, Philadelphia, Pennsylvania; 2 Weill Cornell Medicine, New York, New York; 3 AstraZeneca, Wilmington, Delaware; 4 Milliman, New York, New York; 5 Brigham and Women’s Health and Harvard Medical School, Boston, Massachusetts

**Keywords:** chronic obstructive pulmonary disease, single-inhaler triple therapy, outcomes research, epidemiology, simulation-based projection

## Abstract

**Background:** The US population includes 24 million to 29 million people with diagnosed and undiagnosed chronic obstructive pulmonary disease (COPD). Studies have demonstrated the safety and efficacy of single-inhaler triple therapy (SITT) in reducing COPD exacerbations. Long-term population implications of SITT use have not been quantified.

**Objectives:** This simulation-based projection aimed to estimate the potential impact of widespread SITT use on the US COPD population.

**Methods:** Exacerbation and all-cause mortality reductions reported in the Efficacy and Safety of Triple Therapy in Obstructive Lung Disease trial (ETHOS; NCT02465567) were used to project clinical outcomes in US patients meeting ETHOS trial eligibility criteria (ETHOS-Eligible) and patients meeting a practical definition of SITT eligibility (Expanded ETHOS-Eligible). The US COPD population was modeled with 1000 simulations of patient progression over 10 years. Agent characteristics were based on literature and claims analysis of the 2016-2018 Medicare 100% fee-for-service and IBM MarketScan^®^ databases. Agent annual characteristics reflected incident cases, changes in COPD severity, treatment, mortality, and exacerbations under status quo treatment patterns and scenarios for the adoption of SITT. The scenarios assumed the reduced exacerbation and mortality rates associated with SITT according to ETHOS trial outcomes mean values.

**Results:** Higher than current SITT adoption over 10 years would be expected to substantially reduce COPD exacerbation-associated hospitalizations by 2 million. Applying mean improvements reported in ETHOS for SITT would extend average patient life expectancy 2.2 years for ETHOS-Eligible patients and 1.7 years for Expanded ETHOS-Eligible patients. The number needed to treat to extend the average patient life by 1 year was 8 for the ETHOS-Eligible population and 10 for the Expanded ETHOS-Eligible population.

**Discussion:** Widespread SITT adoption may be impeded by competitive pressures from generic treatments and nonadherence, and efficacy observed in clinical trials may not occur in real-world populations.

**Conclusions:** Assuming ETHOS treatment effects and adherence translate to clinical practice, higher than current use of SITT can substantially reduce COPD exacerbations and hospitalizations and extend survival. These results should be viewed cautiously, because the improved outcomes for SITT in the ETHOS final retrieved vital statistics data were not statistically significant for all comparator therapy groups.

## BACKGROUND

Chronic obstructive pulmonary disease (COPD) is a progressive lung disease impacting approximately 24 million to 29 million US patients, including millions of undiagnosed cases, and is among the leading causes of death in the United States.^1–3^ COPD exacerbations are associated with decreased lung function, declining quality of life, and increased mortality.^4^ Exacerbations are defined as an acute worsening of respiratory symptoms that result in additional therapy and are classified as mild, moderate, and severe.^5^ Moderate exacerbations are classified as those treated with short-acting bronchodilators (SABDs) plus antibiotics and/or oral corticosteroids and severe exacerbations require inpatient hospitalization or an emergency department visit.^6^ In a US study of 17 450 adult patients, 43% of those who were hospitalized for 1 COPD exacerbation in 2009 died within 6 years of the event. That percentage increased to 56% in patients who experienced 2 hospitalizations for COPD.^7^

COPD patients experiencing exacerbations have significantly higher direct medical costs and hospital resource utilization than COPD patients not experiencing exacerbations.^8^ Cumulative direct medical costs in the United States attributed to COPD from 2019 to 2038 are estimated to be $800.9 billion, or $40 billion per year.^9^ Despite being among the leading causes of death, US public funding for COPD research has been low compared with other chronic illnesses.^10^ In 2022, the National Institutes of Health anticipates funding for COPD of $127 million, which is about one-tenth of diabetes funding ($1.25 billion) and less than one-twentieth of cardiovascular funding ($2.62 billion).^11^

Standards of care for patients with COPD include a combination of maintenance inhalers with use of short-acting rescue medications, as well as lifestyle changes, including smoking cessation.^6^ Single-inhaler triple therapy (SITT) combines a long-acting beta agonist (LABA), a long-acting muscarinic antagonist (LAMA), and an inhaled corticosteroid (ICS) in a single inhalation device. ETHOS (NCT02465567) was a 52-week, phase 3, randomized trial to evaluate the efficacy and safety of budesonide/glycopyrrolate/formoterol fumarate (BGF), a SITT, as compared with dual combination therapies, LAMA-LABA and ICS-LABA, in patients with moderate to very severe COPD and at least 1 exacerbation in the prior year.^12,13^ ETHOS results showed a SITT containing 320 µg of budesonide reduced the rate of moderate and severe exacerbations compared with treatment with LAMA-LABA and ICS-LABA therapies by 24% and 13%, respectively.^12^ A predefined secondary endpoint reported in the ETHOS final retrieved dataset showed that the SITT treatment arm had reduced all-cause mortality relative to LAMA-LABA therapy by 49% (hazard ratio [HR] = 0.51, 95% CI = 0.33, 0.80).^13^

The long-term population health outcomes of broader use of SITT have not been quantified. We projected 10-year outcomes of SITT use among US patients with COPD meeting ETHOS clinical trial eligibility criteria (ETHOS-Eligible) and among US patients with COPD meeting a broader, practical definition of eligibility for SITT (Expanded ETHOS-Eligible) more likely applicable to patients in the real world. We aimed to estimate the potential implications of appropriate broader SITT use on the US COPD population if the ETHOS trial results were translated into clinical practice.

## METHODS

### Model Approach

Multi-year stochastic models with patients offer a convenient way to produce ranges of modeled outcomes, model multiple scenarios, and incorporate new entrants (eg, people with newly diagnosed COPD). We used a stochastic model to project health outcomes of the US COPD population over 10 years. In the model, each “Patient” represents an individual COPD patient and is assigned baseline characteristics, such as age, smoking status, COPD stage, and treatment. The Patients were created to represent currently diagnosed COPD patient characteristics in the United States. **Appendix A** describes Patient characteristics and the sources used for the starting year (Year 0) and future years (Year 1+).

COPD patient characteristics were defined using the National Health and Nutrition Survey (NHANES) results. In NHANES, a person with diagnosed COPD is identified by their questionnaire responses as having been told by a doctor they have COPD, emphysema, or current chronic bronchitis. A person with undiagnosed COPD is identified by spirometry, where a respondent’s post-bronchodilator ratio of forced expiratory volume in 1 second (FEV_1_) as a percent of forced vital capacity (FEV_1_/FVC) was at or below 0.7, and the person did not respond to the questionnaire as having been diagnosed. We measured patients’ COPD severity based on the respondent’s percent of predicted FEV_1_. A simulated FEV_1_ measurement was assigned to each Patient when they first appeared in the model, which was based on a COPD severity distribution informed by literature and NHANES along with the Patient’s predicted FEV_1_ based on age, sex, height, and race.

Claims data from 2016-2018 Medicare 100% Research Identifiable Files (RIF) for Medicare fee-for-service beneficiaries and IBM MarketScan® for commercial members was used to develop the current COPD treatment distributions by COPD severity. As spirometry results are not available in claims data, a claims-based algorithm was used to assign severity to each COPD patient.^14^ Patients were mapped to 4 COPD severity ranges, applying a methodology similar to that used in prior claims-based research to establish severity distributions consistent with NHANES spirometry results and literature.^15,16^ In claims databases, we identified moderate exacerbations as those leading to treatment with systemic glucocorticoids, antibiotics, or both for at least 3 days, and severe exacerbations as those resulting in hospitalization or death.

COPD treatment adherence rates were based on the percentage of days covered (PDC) in the claims. For a patient to be considered as being on a treatment, they needed a PDC of at least 25%. A PDC equal to or above 80% was considered adherent and those Patients received the full benefit of treatment. Patients simulated with PDCs between 25% and 80% received a partial treatment benefit using straight-line interpolation between 25% and 80%.

Changes to a Patient’s characteristics were made by applying change factors in a probabilistic manner. A Patient’s COPD severity progressed annually through a decline in FEV_1_. Annual FEV_1_ decline was informed by literature sources and depended on the Patient’s smoking status, COPD severity at the beginning of the year, treatment, and treatment adherence.^17–23^ An additional FEV_1_ decline, also developed from literature, was applied if a patient had a simulated moderate or severe exacerbation during the year.^20^ Severe exacerbations resulted in larger FEV_1_ declines than moderate exacerbations.

Model assumptions were calibrated to achieve the following for a steady-state baseline model:

- All-cause mortality consistent with standard mortality tables adjusted for COPD mortality rates
- Consistent demographic characteristics for the COPD population across the 10 years
- Year-to-year stability of COPD stages for both the existing and incident COPD populations
- COPD treatment patterns that reflect patients’ COPD progression but remain stable over entire COPD population across years

Population growth was added to the steady-state model consistent with 10-year projections of the US population growth to produce the baseline model.^24^

### Simulation Overview, Model Populations, and Simulation Scenarios

The projection modeled 1000 simulations estimating COPD patient outcomes over a 10-year period from 2021 to 2030. Each Patient’s characteristics were set annually based on the likelihood of events, including changes in COPD therapy, COPD exacerbations, changes in FEV_1_ decline dependent on the Patient’s FEV_1_/FVC ratio at the start of the year, and mortality. Over the projection period, new COPD patients entered the model. Mortality was the only exit from the model. All-cause mortality was based on standard age-sex mortality tables adjusted for COPD-related mortality observed in the 2018 Medicare 100% RIF data. If a patient had a severe exacerbation, an additional mortality load was applied, which was derived from 30-day mortality rates following a severe exacerbation observed in the 2018 Medicare 100% RIF data.

The ETHOS trial results were an important source of assumptions. The ETHOS trial found a 320 μg BGF SITT reduced moderate and severe exacerbations by 24% relative to patients taking LAMA-LABA and 13% relative to ICS-LABA.^12^ These findings were applied to patients assigned to SITT.

In the SITT-treated population, mortality risk associated with severe exacerbations was set such that a reduction in severe exacerbations in the modeled ETHOS population reflected the lower mortality rate observed with SITT in the ETHOS trial. A relative risk ratio for all-cause mortality in SITT patients was developed from the mortality observed for ETHOS patients taking 320 μg BGF SITT relative to the LAMA-LABA and ICS-LABA products used in ETHOS. The observed distribution of patients on LAMA-LABA and ICS-LABA in the US ETHOS-Eligible population were used as weights for the relative risk calculation. The applied reduction in mortality was approximately 25% for Patients on SITT relative to Patients not on SITT (**Appendix B**).

The model simulated the current US COPD population of 24 million patients, including 8 million undiagnosed. The COPD population changed annually through deaths and new patients.

Two subsets of the US COPD population were also modeled (See **Appendix C**):

- **ETHOS-Eligible:** This population represents a subset of the US COPD population meeting SITT eligibility criteria from the ETHOS trial.
- **Expanded ETHOS-Eligible**: This population, which is larger than the ETHOS-Eligible population, represents a subset of the US COPD population who may be practical candidates for SITT. Notably, the upper age restriction was removed to include patients over 80 years of age, the smoking status requirement was removed, and lower FEV_1_ values (≥65% of predicted) was allowed in this population.

Three scenarios were modeled for each population:

- **Current (Baseline)**: This scenario models outcomes under the current COPD treatment landscape and considers the impact of current levels of SITT use.
- **Two Higher SITT Adoption (Alternatives)**: These 2 scenarios assume all Patients from Current who meet either the ETHOS-Eligible or Expanded ETHOS-Eligible criteria (defined above) switch to SITT and remain adherent.

We estimated the number of years of life (ie, life-years) extended for the ETHOS-Eligible and Expanded ETHOS-Eligible populations with higher SITT adoption scenarios vs Current. We first calculated expected years of life based on the distribution of age and sex of each Patient using standard mortality tables. We adjusted standard mortality for additional mortality associated with COPD. We estimated expected future years of life for each population.

### Ethics

The use of the Medicare 100% RIF was performed under a waiver of authorization by the Western Institutional Review Board (WIRB Study #1181623; Pr. #20172878). All other data sources utilized were de-identified by the data originator before the authors had access. The use of these de-identified data sources did not require IRB approval, as these were retrospective administrative claims data.

## RESULTS

**Figure 1.**
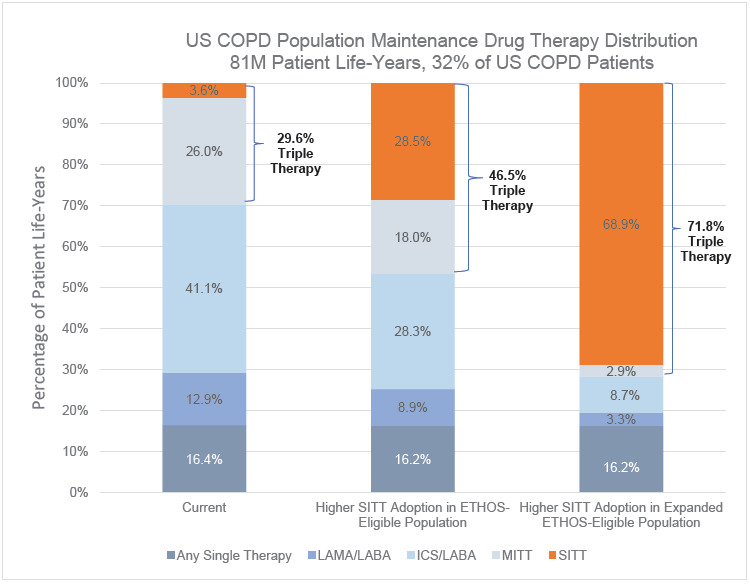
US Maintenance Drug Therapy Distribution Under Current and Higher SITT Adoption Models Abbreviations: COPD, chronic obstructive pulmonary disease; ICS, inhaled corticosteroid; LABA, long-acting beta agonist; LAMA, long-acting muscarinic antagonist; MITT, multiple-inhaler triple therapy; SITT, single-inhaler triple therapy.

Two hundred fifty-five million patient years of life were simulated in the US COPD population from 2021 to 2030. Of the 255 million patient years of life, 79 million were undiagnosed, 95 million were diagnosed and not on maintenance therapy, and 81 million were on a maintenance therapy. Under the Current pharmacological drug distribution, 29.6% of the US COPD population on maintenance therapy were treated with multiple-inhaler triple therapy (MITT) or SITT. The MITT or SITT treatment rate was 46.5% when applying the ETHOS-Eligible population and 71.8% when applying the Expanded ETHOS-Eligible population. The higher SITT adoption scenario shifted patients predominantly from either LAMA-LABA, ICS-LABA, or MITT to SITT, while leaving the distribution of treatments for patients with lower severity stable. The full distribution of COPD maintenance drug therapies in the US COPD population is shown in Figure 1.

**Figure 2.**
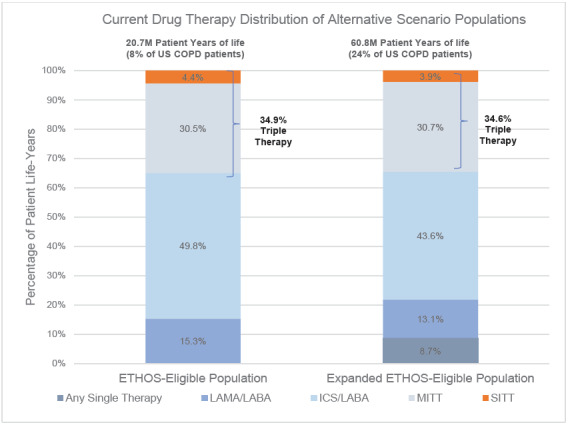
ETHOS-Eligible and Expanded ETHOS-Eligible Drug Therapy Distribution Under Current Abbreviations: COPD, chronic obstructive pulmonary disease; ICS, inhaled corticosteroid; LABA, long-acting beta agonist; LAMA, long-acting muscarinic antagonist; MITT, multiple-inhaler triple therapy; SITT, single-inhaler triple therapy.

Under the Current drug therapy distribution, 4.4% of the ETHOS-Eligible and 3.9% of the Expanded ETHOS-Eligible patients were on SITT. The higher SITT adoption scenario moved each SITT-eligible patient to SITT usage, and in this setting, the Expanded ETHOS-Eligible population was nearly 3 times the size of the ETHOS-Eligible population. Besides the inclusion of patients on a single maintenance therapy who also had a severe exacerbation (which was <10% of all patients), the current drug therapy distribution between both populations was similar. This indicates the Expanded ETHOS-Eligibility criteria identified triple therapy–eligible COPD patients instead of predominantly lower or higher acuity patients. Figure 2 shows the Current COPD drug maintenance therapy distribution of the ETHOS-Eligible and Expanded ETHOS-Eligible patient populations.

Under Current, the average SITT PDC was 65%, which our interpolation algorithm converts to producing 73% of the treatment benefits of the ETHOS trial. After increasing the adherence of Patients on SITT to 80% (full adherence), annual severe exacerbations dropped by 6.7% (from 0.25 to 0.23), annual moderate exacerbations dropped by 6.4% (from 0.93 to 0.87), and annual all-cause mortality decreased by 0.4% (from 3.13% to 3.11%).

Across the US COPD population, ETHOS-Eligible population, and Expanded ETHOS-Eligible populations, the payer distribution was similar, with Medicare being the majority, followed by commercial, and then the Medicaid population. The US COPD population had a small majority of females, and the ETHOS-Eligible population was nearly evenly split by sex, whereas the Expanded ETHOS-Eligible population was nearly 60% female. The average age of US COPD patients was about 68 years old, whereas the ETHOS-Eligible population was about 1 year younger and the Expanded ETHOS-Eligible was about 1 year older. The US COPD and Expanded ETHOS-Eligible populations had a similar distribution of smoking status, consisting predominantly of former smokers, followed by current smokers and nonsmokers, whereas the ETHOS-Eligible population excluded nonsmokers. The US COPD population’s severity distribution was consistently spread between FEV_1_ ranges above 30% as a percentage of predicted, but both ETHOS-Eligible populations were concentrated in higher severity patients and had no lowest severity patients. Table 1 provides detailed demographic information under all scenarios for each population.

**Table 1.** Population Demographics and COPD Severity Distribution Over 10 Years by Scenario

	**Total US COPD Population**	**ETHOS-⁠Eligible Population**		**Expanded ETHOS-⁠Eligible Population**	
	**Current**	**Current**	**Higher SITT Adoption**	**Current**	**Higher SITT Adoption**
Patient outcomes—cumulative					
Total years of life (millions)	254.7	20.7	21.5	60.8	63.3
Population demographics					
By payer					
Commercial, %	23	22	22	20	20
Medicaid, %	14	16	16	14	14
Medicare, %	63	62	63	66	66
By sex					
Female, %	53	49	49	59	59
Average age, end of year	68.3	67.3	67.3	69.5	69.5
Average smoking status, end of year					
Current, %	33	40	40	31	30
Former, %	48	60	60	51	51
Never, %	20	0	0	19	19
FEV_1_ distribution, end of year					
FEV_1_ >80% predicted, %	27	0	0	0	0
FEV_1_ ≥50% and <80% predicted, %	39	10	10	9	9
FEV_1_ ≥30% and <50% predicted, %	27	72	72	64	64
FEV_1_ <30% predicted, %	8	18	18	27	26

Abbreviations: COPD, chronic obstructive pulmonary disease; FEV_1_, forced exhalation volume in 1 second; SITT, single-inhaler triple therapy.

Patient exacerbation and all-cause mortality outcomes varied across each scenario and population. Higher SITT Adoption led to lower exacerbation rates (both severe and moderate) and lower all-cause mortality.

Under Higher SITT Adoption, the proportion of years of life with at least 1 severe exacerbation for the ETHOS-Eligible population dropped from 22.5% to 19.5%, a 13.2% reduction, and for the Expanded ETHOS-Eligible population from 24.3% to 21.1%, a 13.0% reduction. This reduction in ETHOS-Eligible severe exacerbations represents an overall reduction of 1.6% in US COPD severe exacerbations and the reduction from the Expanded ETHOS-Eligible represents an overall reduction of 5.0% in US COPD severe exacerbations.

Under Higher SITT Adoption relative to the Current, the all-cause mortality rate for the ETHOS-Eligible population fell from 4.0% to 3.0%, a 25.5% reduction, and for the Expanded ETHOS-Eligible population from 5.4% to 4.3%, a 19.6% reduction. These reductions in all-cause mortality in the ETHOS-Eligible group and Expanded ETHOS-Eligible population represents an overall reduction of all-cause mortality in the US COPD population of 2.0% and 5.8%, respectively, over the 10-year projection period. Table 2 provides patient outcome results for all-cause mortality and exacerbations based on patient years of life.

**Table 2.** Population Exacerbation All-Cause Mortality Outcomes by Population and Scenario Over 10 Years

	**Total US COPD Population**			**ETHOS-Eligible Population**		**Expanded ETHOS- Eligible Population**	
	**Current**	**Higher SITT Adoption: ETHOS- Eligible Population**	**Higher SITT Adoption: Expanded ETHOS-Eligible Population**	**Current**	**Higher SITT** **Adoption**	**Current**	**Higher SITT** **Adoption**
Total years of life (millions)	254.7	255.5	257.2	20.7	21.5	60.8	63.3
Severe exacerbations (millions)	38.7	38.1	36.7	5.4	4.8	17.4	15.4
Moderate exacerbations (millions)	202.2	199.6	194.4	21.4	18.8	65	57.1
Deaths (millions)	10.7	10.5	10.2	0.8	0.7	3.3	2.8
≥1 severe exacerbation (%)	13.5	13.3	12.9	22.5	19.5	24.3	21.1
≥1 moderate exacerbation (%)	53.5	53.0	52.1	63.9	57.9	65.0	59.0
All-⁠cause mortality rate (%)	4.2	4.1	3.9	4.0	3.0	5.4	4.3
All-cause mortality rate on triple therapy (%)	4.6	4.0	4.3	3.1	3.0	4.7	4.3

Abbreviations: COPD, chronic obstructive pulmonary disease; FEV_1_, forced exhalation volume in 1 second; SITT, single-inhaler triple therapy.

**Figure 3.**
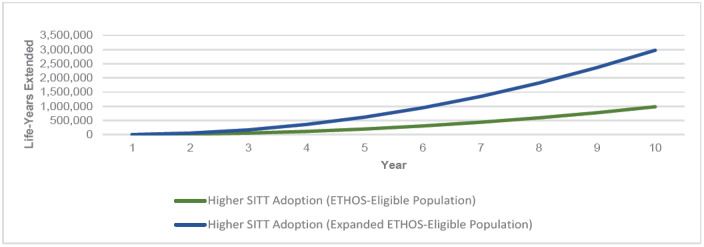
Cumulative Years of Life Extended Under Higher SITT Adoption Scenarios for the US COPD Population Abbreviation: SITT, single-inhaler triple therapy.

Higher SITT Adoption in both the ETHOS-Eligible and Expanded ETHOS-Eligible populations consistently had fewer all-cause deaths per year and cumulatively over the 10-year simulation. As the COPD population grew over time, so did the annual number of deaths across each population. However, Higher SITT Adoption scenarios each led to fewer annual deaths and increased cumulative years of life extended. **Appendix D** illustrates the annual all-cause deaths in the US COPD population under each scenario. Figure 3 illustrates the cumulative years of life extended in the US COPD population under Higher SITT Adoption relative to Current.

We measured a range of the impact of Higher SITT Adoption by developing a distribution of the difference from each Patient outcome between the Current and Higher SITT Adoption scenarios. We found that the Higher SITT Adoption scenario extended the ETHOS-Eligible population by nearly 1 million years of life and the Expanded ETHOS-Eligible population by nearly 3 million years of life over the 10-year projection period. For the ETHOS-Eligible and Expanded ETHOS-Eligible populations, we estimated that the Current future life expectancy was 16 and 17 years, respectively. Higher SITT Adoption would extend the average patient life by 2.2 years for ETHOS-Eligible patients and 1.7 years for Expanded ETHOS-Eligible patients. The fewer years of life added for the latter population may in part be driven by their higher average age (69.5 vs 67.3).

We estimated the number needed to treat as the number of patients on SITT annually to extend that patient group’s expected years of life by 1 year on average. We estimated that for the ETHOS-Eligible population 8 patients on SITT annually would extend a year of life to 1 patient each year on average, and for the Expanded ETHOS-Eligible population, 10 patients on SITT annually would extend a year of life to 1 patient each year on average. Detailed results on the impact of Higher SITT Adoption over Current, including the 5th and 95th percentile of ranges, are shown in Table 3.

**Table 3.** Years of Life Extended and Exacerbation Reduction Under Higher SITT Adoption Over 10 Years

**Metric**	**ETHOS-Eligible Population**		**Expanded ETHOS-Eligible Population**	
	**Average (5th-⁠95th Percentile)**	**% of Total, Average (5th-⁠95**th **Percentile)**	**Average (5th-⁠95th Percentile)**	**% of Total, Average (5th-⁠95th Percentile)**
Cumulative years of life extended (millions)	0.983 (0.700, 1.355)	4.8 (3.4, 6.6)	2.976 (2.564, 3.485)	5.1 (4.4, 5.9)
Severe exacerbations avoided per year per patient	0.039 (0.032, 0.046)	14.9 (12.3, 17.7)	0.042 (0.039, 0.046)	15.3 (14.1, 16.6)
Moderate exacerbations avoided per year per patient	0.159 (0.145, 0.173)	15.4 (14.0, 16.8)	0.164 (0.157, 0.172)	15.7 (14.9, 16.3)
All-cause mortality reduction, %	1.0 (0.7, 1.4)	25.5 (18.2, 35.5)	1.0 (0.9, 1.2)	20.7 (17.8, 24.3)
Years of life added per patient	2.22 (0.74, 4.08)	14.3 (4.8, 26.2)	1.72 (1.12, 2.36)	10.3 (6.7, 14.2)
No. needed to treat^a^	8 (21, 4)	n/a	10 (15, 8)	n/a

Abbreviation: NA, not available; SITT, single-inhaler triple therapy.

^a^Number needed to treat is an annual measure of the number of ETHOS-Eligible or Expanded ETHOS-Eligible COPD patients on SITT for 1 year to extend at least 1 patient’s total year of life by 1 on average.

The historical average PDC was 65%. After increasing the adherence of those on SITT from 65% (partial adherence) to 80% (full adherence) we found that adherence resulted in an approximately 6% decrease in total exacerbations and less than 0.5% decrease in mortality among COPD patients on SITT.

## DISCUSSION

This analysis evaluates the potential application of SITT in the real world assuming that treatment effects observed in the ETHOS trial were applied to the population of US patients with COPD. These results should be viewed cautiously, because the improved outcomes for SITT in the ETHOS final retrieved vital statistics data were not statistically significant for all comparator therapy groups.^13^ Assuming mean reported outcomes from the ETHOS trial, higher than current adoption of SITT over 10 years would be expected to substantially reduce exacerbation-associated hospitalizations and extend average patient life expectancy by 2.2 years for ETHOS-Eligible patients and 1.7 years for Expanded ETHOS-Eligible patients. The number needed to treat to extend the average patient life by 1 year was estimated as 8 for the ETHOS-Eligible population and 10 for the Expanded ETHOS-Eligible population. The potential large gains in exacerbation and mortality reduction from application of SITT in this study can be additive to other strategies to improve COPD outcomes, such as guideline-concordant comprehensive management, smoking cessation, supplemental oxygen therapy, and pulmonary rehabilitation. The ETHOS trial demonstrated better health outcomes with SITT vs dual maintenance therapies with similar adherence rates; however, in the real world, adherence to maintenance treatment is generally low in patients with COPD.^25^

A post-hoc analysis from the ETHOS trial found the SITT, BGF, reduced the risk of all-cause mortality compared with glycopyrrolate/formoterol fumarate by 49% (HR, 0.51; 95% CI, 0.33-0.80) but did not achieve a statistical significant reduction compared with budesonide/formoterol fumarate (HR, 0.72; 95% CI, 0.44-1.16) over 52 weeks in the final retrieved data set.^13^ While the outcomes from one trial, ETHOS, were applied in this model, the IMPACT trial found, for fluticasone furoate/umeclidinium/vilanterol (a SITT), a 28% reduction in death (HR, 0.72; 95% CI, 0.53-0.99; *P* = .042) vs umeclidinium/vilanterol (a LABA/LAMA).^26^ Both ETHOS and IMPACT found mortality impacts of COPD therapy, which may add credibility to the assumptions used in this modeling.

The ETHOS trial findings were applied to both the ETHOS-Eligible and a broader Expanded ETHOS-Eligible populations. The Expanded ETHOS-Eligible scenario relaxed some of the exclusions of more severe or older COPD patients. In particular, we relaxed the ETHOS upper age limit and the lower bound on FEV_1_ as a percentage of predicted, as well as requirements for smoking history and prior COPD treatment. The Expanded ETHOS-Eligible population used in the model was based on claims history and may not identify all patients who could benefit from SITT, because patients not treated with maintenance therapies may not be included in the Expanded ETHOS-Eligible population.

The data compiled while building the model came from many sources and several years of data. As with other long-term simulations, numerous modeling assumptions were used, and other choices of assumptions could produce different results. NHANES results were used to define COPD patient characteristics; notably, these data omit the institutionalized population and therefore patients in nursing facilities are not distinguished from those in the community setting. It is unclear whether the effects of SITT would be different in patients in different care settings. Achieving the higher SITT use assumed in the model would require changes to clinical practice and perhaps public health efforts. Our models assumed mean ETHOS results, including comparison to budesonide/formoterol fumarate, which were not statistically significant.

In an ideal scenario, in which all Expanded ETHOS-Eligible patients shift to SITT, take SITT as prescribed, and treatment efficacy from observed ETHOS clinical trial results is maintained, SITT would then prevent 2 million COPD hospitalizations and add 3 million years of life to the US population over the next 10 years. Full adoption and adherence to SITT among Expanded ETHOS-Eligible patients is unlikely to be realized. Broad SITT adoption may be impeded by competitive market pressures of generic treatments on the market as well as nonadherence, and efficacy observed in clinical trials may not occur in real-world populations. The model results depend on 2 key assumptions: the effectiveness of SITT and the uptake of SITT. As a sensitivity to these considerations, we note that if SITT adoption were used for only 20% of the broader population identified as Expanded ETHOS-Eligible patients, and the effect in ETHOS were reduced by 50%, the use of SITT could still avoid 200 000 COPD hospitalizations and add 300 000 years of life to the US population over 10 years. The results of this 20% × 50% sensitivity are 10% of the Expanded ETHOS eligible scenario, which suggest that, as expected, useful sensitivities to the 2 key assumptions can be obtained through linear interpolations.

Assuming trial-derived mean treatment effects hold in a real-world population would be considered ideal; however, achieving such treatment results over a 10-year period at the population level would be difficult. The findings from this simulation-based projection study of the ETHOS trial to a real-world US COPD population provide rationale for dedicated strategies to consider implementation of SITT among appropriate treatment-eligible patients. The potential reduction in mortality on a population level points to the need for a definitive understanding of methods to reduce COPD mortality.

### Author Contributions

B.P. was involved with concept development and design of the study, data acquisition, analysis and interpretation of the data, and developing drafts and revisions of the manuscript. G.C. was involved with concept development and design of the study, analysis and interpretation of the data and developing drafts and revisions of the manuscript. F.M. was involved with concept development and design of the study, analysis and interpretation of the data and developing drafts and revisions of the manuscript. H.G. was involved with concept development and design of the study, analysis and interpretation of the data and developing drafts and revisions of the manuscript. N.F. was involved with concept development and design of the study and developing drafts and revisions of the manuscript. M.E. was involved with concept development and design of the study, data acquisition, analysis and interpretation of the data, and developing drafts and revisions of the manuscript. U.G. was involved with concept development and design of the study, data acquisition, analysis and interpretation of the data, and developing drafts and revisions of the manuscript. M.V. was involved with concept development and design of the study, analysis and interpretation of the data and developing drafts and revisions of the manuscript.

### Disclosures

Milliman (which employed coauthors B.P., M.E., and U.G.) received consulting fees from AstraZeneca. H.G. and N.F. are employees and shareholders of AstraZeneca. M.V. has received research grant support or served on advisory boards for American Regent, Amgen, AstraZeneca, Bayer AG, Baxter Healthcare, Boehringer Ingelheim, Cytokinetics, Lexicon Pharmaceuticals, Novartis, Pharmacosmos, Relypsa, Roche Diagnostics, Sanofi, and Tricog Health and speaker engagements with AstraZeneca, Novartis, and Roche Diagnostics, and participates on clinical trial committees for studies sponsored by Galmed, Novartis, Bayer AG, Occlutech, and Impulse Dynamics. F.M. has served on advisory boards for AstraZeneca, Boehringer Ingelheim, Chiesi, GlaxoSmithKline, Novartis, Sanofi /Regeneron, and Teva; steering committees for AstraZeneca, Chiesi, and GlaxoSmithKline; and a Data and Safety monitoring board for GlaxoSmithKline; and has provided teleconsultation for Bayer outside the submitted work. G.C. received grants from NIH-NHLBI, PA-DOH, GSK, Boehringer-Ingelheim, Novartis, AstraZeneca, Respironics, MedImmune, Novartis, Pearl, PneumRx, Pulmonx, Broncus, Spiration, Olympus, Fisher-Paykel Healthcare, Chiesi, Gilead, Pfizer, Corvus, Lilly, Regeneron, Genetech, Roche. Consultant for: Amirall, AstraZeneca, Nuvaira, GSK, CSA Medical, PneumRX, BTG, Mereo, Broncus, Pulmonx, GSK, EOLO.

### Data Availability

Due to contractual restrictions imposed by the database providers, the source datasets are not available.

## Supplementary Material

Online Supplementary Material
