# Supplementary material for: PROMETHEUS: Long-Term Exacerbation and Mortality Benefits of Implementing Single-Inhaler Triple Therapy in the US COPD Population
Source: J Health Econ Outcomes Res. 2023 Jan 24;10(1):20–7. doi: 10.36469/001c.55635 (PMC9879267; doi:10.36469/001c.55635)
Supplement: Online Supplementary Material [file jheor_2023_10_1_55635_135822.pdf]

Journal of Health Economics and Outcomes Research

Online Supplementary Material

PROMETHEUS: Long-Term Exacerbation and Mortality Benefits of Implementing Single-Inhaler Triple Therapy in the US COPD Population. *JHEOR*. **2023;10(1):20-27.** doi:10.36469/jheor.2023.55635

Appendix A: Patient Characteristics Sources

Appendix B: Original ETHOS Outcomes (Time to Death)

Appendix C: Description of ETHOS Eligibility Criteria

Appendix D: Average Deaths per Year by Scenario for the US COPD Population

Appendix E: Model Calibration Adjustments

Appendix F: Patient Exacerbation Year of Life Outcomes

Appendix G: Stochastic Variable Detail

This supplementary material has been provided by the authors to give readers additional information about their work.

This is an open-access article distributed under the terms of the Creative Commons Attribution 4.0 International License (CCBY-4.0). View this license's legal deed at http://creativecommons.org/licenses/by/4.0 and legal code at http://creativecommons.org/licenses/by/4.0/legalcode for more information.

Appendix A: Patient Characteristics Sources

CHARACTERISTICS	SOURCE FOR YEAR 0	SOURCE FOR YEAR 1+
Age	NHANES 2007-2008 through 2017-2018[14]	Increase by 1 each year
Sex	NHANES 2007-2008 through 2017-2018	Fixed
Smoking status	NHANES 2007-2008 through 2017-2018	NIH PATH study (2015)[1] and Jiménez-Ruiz (2001)[16] inform smoking cessation or initiation rates.
Payer	NHANES 2017-2018	Assume fixed, except for transitions into Medicare at age 65
Predicted FEV_1	Hankinson et al (1999)[17] predicted FEV_1 reference tables using a patient's age, sex, height, and race developed from NHANES 2007-2008 through 2017-2018.	Hankinson et al (1999)[17] predicted FEV_1 reference tables using a patient's age, sex, height, and race.
FEV_1 as a % of predicted	Initial COPD severity distribution informed by analysis of NHANES spirometry results from 2007-2008 through 2011-2012, Hernández et al (2018)[18] and Wallace et al (2019)[19]	FEV_1 declines annually based on smoking status and the patient's FEV_1 ratio at the start of the year based on Bhatt et al (2016),[20] Vestbo & Lange (2016),[21] and Lee & Fry (2010).[22] An additional decline in FEV_1 is applied if the patient had an exacerbation during the year based on Dransfield et al (2017).[23]
Moderate/severe exacerbation(s)	Hurst et al (2010)[24]	Hurst et al (2010)[24] (with SITT reducing exacerbations based on the ETHOS trial)
COPD treatment and adherence	2016-2018 Medicare 100% FFS and IBM MarketScan databases for diagnosed patients.	Treatment subject to change in year after COPD severity changes and adherence subject to change annually
Asthma diagnosis	NHANES 2007-2008 through 2017-2018	Same

Abbreviation: FEV_1, forced exhalation volume (volume of air a patient can exhale during a forced breath in 1 second and obtained from a spirometry exam.

Appendix B: Original ETHOS Outcomes (Time to Death)

ORIGINAL ETHOS OUTCOMES (TIME TO DEATH)			
**DEVELOPMENT OF MORTALITY BENEFIT FOR AGENT BASED MODELING**			
	**BGF 320-µg**	**LAMA/LABA**	**ICS/LABA**
US population–weighted ETHOS distribution	27%	17%	56%
ETHOS mortality outcomes	1.3%	2.3%	1.6%
% improvement (relative risk ratio)	NA	43%	19%
Weighted-average % improvement[a]	NA	**24.5%**	

Abbreviations: BGF, budesonide/glycopyrrolate/formoterol fumarate; ICS, inhaled corticosteroid; LABA, long-acting beta agonist; LAMA, long-acting muscarinic antagonist.

[a]Calculated as the weighted average % improvement using the US population weighted–ETHOS distribution.

Appendix C: Description of ETHOS Eligibility Criteria

CRITERION	ETHOS-ELIGIBLE	EXPANDED ETHOS-ELIGIBLE
Age (y)	40-80	40+
Smoker status	Current or former smoker with 10 pack-year history	No requirement
Exacerbations	At least 1 prior modeled exacerbation	At least 1 prior modeled exacerbation
FEV_1 as a % of predicted	FEV_1 is between 25% - 65% of predicted FEV_1	FEV_1 is at or below 65% of predicted FEV_1
COPD treatment	At least 2 inhaled maintenance therapies	At least 2 inhaled maintenance therapies, or 1 inhaled maintenance therapy and a prior severe exacerbation
Asthma	Excludes patients diagnosed with asthma	No exclusion

Abbreviations: COPD, chronic obstructive pulmonary disease; FEV_1, forced exhalation volume in 1 second.

Appendix D: Average Deaths per Year by Scenario for the US COPD Population

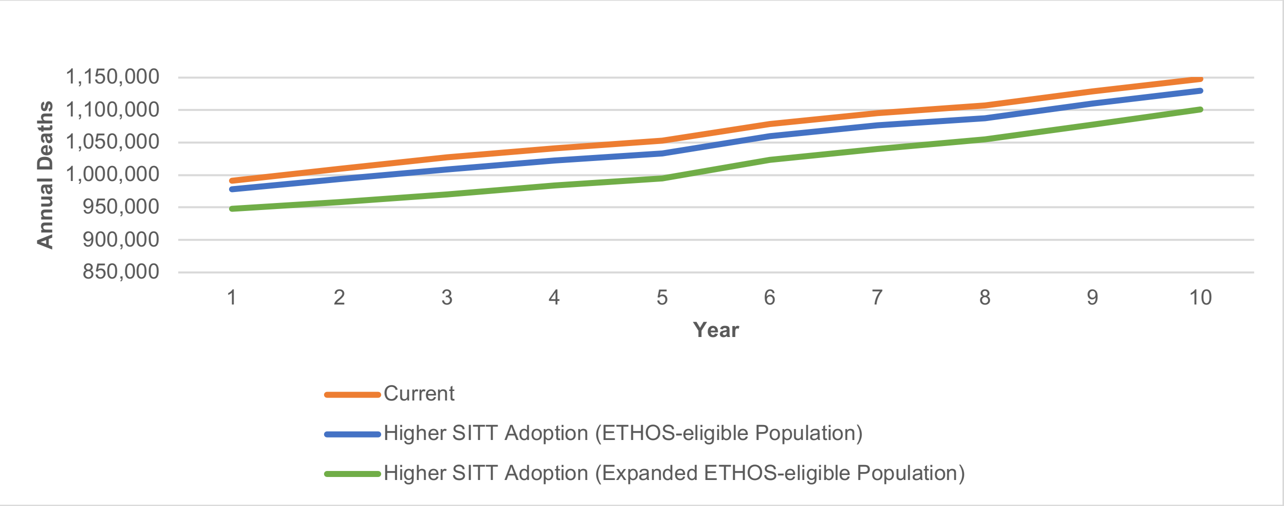

Abbreviations: COPD, chronic obstructive pulmonary disease; SITT, single-inhaler triple therapy.

Appendix E: Model Calibration Adjustments

We made the following adjustments to achieve a steady-state baseline model:

- Set number of new entrants (incident COPD population) to achieve a stable COPD population over time
- Reduced average age of new entrants to reflect a bolus of new entrants at age 40 years (consistent with models' earliest age being 40 years)
- Adjusted mortality rates by COPD severity for new entrants to produce stable all-cause mortality and COPD progression across years
- Adjusted FEV_1 annual decline by age reflect younger COPD patients having higher FEV_1 than older COPD patients and to reflect COPD severity consistent with literature

Appendix F: Patient Exacerbation Year of Life Outcomes

	Total US COPD Population			ETHOS-Eligible Population		Expanded ETHOS-Eligible Population	
	Current	Higher SITT Adoption – ETHOS-Eligible Population	Higher SITT Adoption – Expanded ETHOS-Eligible Population	Current	Higher SITT Adoption	Current	Higher SITT Adoption
Severe exacerbations per year per patient	0.15	0.15	0.14	0.26	0.22	0.29	0.24
Severe exacerbations on triple therapy per year per patient	0.30	0.26	0.24	0.27	0.22	0.31	0.24
Moderate exacerbations per year per patient	0.79	0.78	0.76	1.03	0.88	1.07	0.90
Moderate exacerbations on triple therapy per year per patient	1.01	0.94	0.90	0.99	0.88	1.04	0.90

Abbreviations: COPD, chronic obstructive pulmonary disease; SITT, single-inhaler triple therapy.

Appendix G: Stochastic Variable Detail

Variable	Description	Frequency	Distribution	Formula *f(x)**
Starting age	Patient's age when entering the model	Once, prior to entering model	Discrete uniform, within age bands	1/(Age Max - Age Min)
Height	Patient's height, does not change throughout simulation	Once, prior to entering model	Discrete uniform, within height bands	1/(Height Max - Height Min)
Starting FEV_1	Patient's FEV_1 when entering the model, either in the initial COPD population or as a new entrant	Once, prior to entering model	Continuous uniform, within COPD severity stage	1/(FEV_1 Max - FEV_1 Min)
Undiagnosed status	Patient's COPD undiagnosed status (only mild/moderate COPD stage agents)	Once, prior to entering model	Binomial distribution with mean as probability of being undiagnosed	*p*(Undiagnosed | COPD Severity Mild or Moderate)*(1 - *p*(Undiagnosed | COPD Severity Mild or Moderate)
Diagnosed status	If a patient is undiagnosed, determine if they should be diagnosed	When a patient is undiagnosed and their COPD stage changes	Binomial distribution with mean as probability of being diagnosed. Patients who had a severe exacerbation in the year are considered diagnosed automatically.	p(Diagnosed | COPD Severity)* [1-p(Diagnosed | COPD Severity)]
Drug therapy	If a patient is diagnosed, identify which type of drug therapy they are taking	At model entry and at change in COPD severity level	Discrete random variable Possible drugs are ICS, LAMA, LABA, ICS-LABA, LAMA-LABA, MITT, SITT, or None. Distribution based on observed distributions in claims data.	*p*(Drug Treatment | Previous Drug Treatment, COPD Severity, and Payer Type)
Adherence	Patient's proportion of days covered, if they are taking a drug therapy	Annually as long as a patient is on a drug therapy	Continuous uniform between 25 (minimum adherence) and 80 (full adherence)	1/(80 - 25)
No. of severe exacerbations	Patient's number of severe exacerbations over 1 year	Annually	Poisson random variable with mean based on observed rates in claims data. Adjustments to mean made on drug therapy and adherence.	Poisson [(Mean Severe Exacerbations | COPD Severity) * Impact of Triple Therapy * Impact of Adherence]
No. of moderate exacerbations	Patient's number of moderate exacerbations over 1 year	Annually	Poisson random variable with mean based on observed rates in claims data. Adjustments to mean made on drug therapy and adherence.	Poisson [(Mean Moderate Exacerbations | COPD Severity) * Impact of Triple Therapy * Impact of Adherence]

Variable	Description	Frequency	Distribution	Formula *f(x)**
Change in smoking status	Patient's smoking status for year	Annually	Discrete random variable. Probability varies based on patient's current smoking status. Eligible smoking statuses are Never, Current, or Former	*p*(Smoking Status | Previous Smoking Status)
FEV_1 decay	FEV_1 decay rate for patients	Annually	Normally distributed with mean and standard deviation based on COPD severity and smoking status. If FEV_1 decay is positive, set decay to 0. Additional decay added if the patient had exacerbation in year based on stage	Normal(mean | COPD Severity and Smoking Status, standard deviation | COPD Severity and Smoking Status) + p(1 or more exacerbation)*(decay due to exacerbation | COPD Severity)
Mortality	Did the patient die in the year?	Annually	Binomial distribution with mean as probability of dying subject to age and sex. If the patient does not die due to their age/sex mortality, Patients may also die if they had a severe exacerbation in the year.	Age Sex Death: *p*(death) * (1-*p*(death))<br><br>Exacerbation Death (if no Age/Sex Death): *p*(Death from Severe Exacerbation)) ^ No. of Severe Exacerbations

Abbreviations: Abbreviations: COPD, chronic obstructive pulmonary disease; FEV_1, forced exhalation volume in 1 second; ICS, inhaled corticosteroid; LABA, long-acting beta agonist; LAMA, long-acting muscarinic antagonist; MITT, multiple-inhaler triple therapy; SITT, single-inhaler triple therapy.

**p(x)* = probability of *x*, eg, *p*(death) = annual probability a patient dies; *p(x | y*) = probability of *x* given *y*, eg, *p*(Drug Treatment | COPD Severity) = annual probability of drug a treatment given a patient's COPD severity.

Appendix A: Patient Characteristics Sources

CHARACTERISTICS	SOURCE FOR YEAR 0	SOURCE FOR YEAR 1+
Age	NHANES 2007-2008 through 2017-2018[14]	Increase by 1 each year
Sex	NHANES 2007-2008 through 2017-2018	Fixed
Smoking status	NHANES 2007-2008 through 2017-2018	NIH PATH study (2015)[1] and Jiménez-Ruiz (2001)[16] inform smoking cessation or initiation rates.
Payer	NHANES 2017-2018	Assume fixed, except for transitions into Medicare at age 65
Predicted FEV_1	Hankinson et al (1999)[17] predicted FEV_1 reference tables using a patient's age, sex, height, and race developed from NHANES 2007-2008 through 2017-2018.	Hankinson et al (1999)[17] predicted FEV_1 reference tables using a patient's age, sex, height, and race.
FEV_1 as a % of predicted	Initial COPD severity distribution informed by analysis of NHANES spirometry results from 2007-2008 through 2011-2012, Hernández et al (2018)[18] and Wallace et al (2019)[19]	FEV_1 declines annually based on smoking status and the patient's FEV_1 ratio at the start of the year based on Bhatt et al (2016),[20] Vestbo & Lange (2016),[21] and Lee & Fry (2010).[22] An additional decline in FEV_1 is applied if the patient had an exacerbation during the year based on Dransfield et al (2017).[23]
Moderate/severe exacerbation(s)	Hurst et al (2010)[24]	Hurst et al (2010)[24] (with SITT reducing exacerbations based on the ETHOS trial)
COPD treatment and adherence	2016-2018 Medicare 100% FFS and IBM MarketScan databases for diagnosed patients.	Treatment subject to change in year after COPD severity changes and adherence subject to change annually
Asthma diagnosis	NHANES 2007-2008 through 2017-2018	Same

Abbreviation: FEV_1, forced exhalation volume (volume of air a patient can exhale during a forced breath in 1 second and obtained from a spirometry exam.

Appendix B: Original ETHOS Outcomes (Time to Death)

ORIGINAL ETHOS OUTCOMES (TIME TO DEATH)			
**DEVELOPMENT OF MORTALITY BENEFIT FOR AGENT-BASED MODELING**			
	**BGF 320-µg**	**LAMA/LABA**	**ICS/LABA**
US population–weighted ETHOS distribution	27%	17%	56%
ETHOS mortality outcomes	1.3%	2.3%	1.6%
% improvement (relative risk ratio)	NA	43%	19%
Weighted-average % improvement[a]	NA	**24.5%**	

Abbreviations: BGF, budesonide/glycopyrrolate/formoterol fumarate; ICS, inhaled corticosteroid; LABA, long-acting beta agonist; LAMA, long-acting muscarinic antagonist.

[a]Calculated as the weighted average % improvement using the US population weighted–ETHOS distribution.

Appendix C: Description of ETHOS Eligibility Criteria

CRITERION	ETHOS-ELIGIBLE	EXPANDED ETHOS-ELIGIBLE
Age (y)	40-80	40+
Smoker status	Current or former smoker with 10 pack-year history	No requirement
Exacerbations	At least 1 prior modeled exacerbation	At least 1 prior modeled exacerbation
FEV_1 as a % of predicted	FEV_1 is between 25% - 65% of predicted FEV_1	FEV_1 is at or below 65% of predicted FEV_1
COPD treatment	At least 2 inhaled maintenance therapies	At least 2 inhaled maintenance therapies, or 1 inhaled maintenance therapy and a prior severe exacerbation
Asthma	Excludes patients diagnosed with asthma	No exclusion

Abbreviations: COPD, chronic obstructive pulmonary disease; FEV_1, forced exhalation volume in 1 second.

Appendix D: Average Deaths per Year by Scenario for the US COPD Population

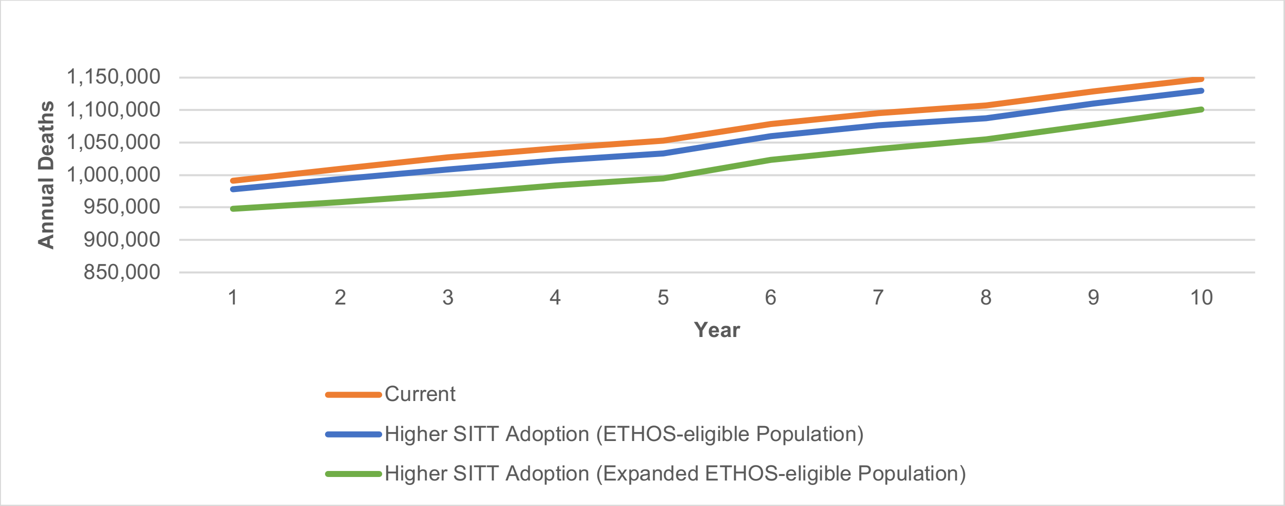

Abbreviations: COPD, chronic obstructive pulmonary disease; SITT, single-inhaler triple therapy.

Appendix E: Model Calibration Adjustments

We made the following adjustments to achieve a steady-state baseline model:

- Set number of new entrants (incident COPD population) to achieve a stable COPD population over time
- Reduced average age of new entrants to reflect a bolus of new entrants at age 40 years (consistent with models' earliest age being 40 years)
- Adjusted mortality rates by COPD severity for new entrants to produce stable all-cause mortality and COPD progression across years
- Adjusted FEV_1 annual decline by age reflect younger COPD patients having higher FEV_1 than older COPD patients and to reflect COPD severity consistent with literature

Appendix F: Patient Exacerbation Year of Life Outcomes

	Total US COPD Population			ETHOS-Eligible Population		Expanded ETHOS-Eligible Population	
	Current	Higher SITT Adoption – ETHOS-Eligible Population	Higher SITT Adoption – Expanded ETHOS-Eligible Population	Current	Higher SITT Adoption	Current	Higher SITT Adoption
Severe exacerbations per year per patient	0.15	0.15	0.14	0.26	0.22	0.29	0.24
Severe exacerbations on triple therapy per year per patient	0.30	0.26	0.24	0.27	0.22	0.31	0.24
Moderate exacerbations per year per patient	0.79	0.78	0.76	1.03	0.88	1.07	0.90
Moderate exacerbations on triple therapy per year per patient	1.01	0.94	0.90	0.99	0.88	1.04	0.90

Abbreviations: COPD, chronic obstructive pulmonary disease; SITT, single-inhaler triple therapy.

Appendix G: Stochastic Variable Detail

**Variable**	**Description**	**Frequency**	**Distribution**	**Formula *f(x)****
Starting age	Patient's age when entering the model	Once, prior to entering model	Discrete uniform, within age bands	1/(Age Max - Age Min)
Height	Patient's height, does not change throughout simulation	Once, prior to entering model	Discrete uniform, within height bands	1/(Height Max - Height Min)
Starting FEV_1	Patient's FEV_1 when entering the model, either in the initial COPD population or as a new entrant	Once, prior to entering model	Continuous uniform, within COPD severity stage	1/(FEV_1 Max - FEV_1 Min)
Undiagnosed status	Patient's COPD undiagnosed status (only mild/moderate COPD stage agents)	Once, prior to entering model	Binomial distribution with mean as probability of being undiagnosed	*p*(Undiagnosed | COPD Severity Mild or Moderate)*(1 - *p*(Undiagnosed | COPD Severity Mild or Moderate)
Diagnosed status	If a patient is undiagnosed, determine if they should be diagnosed	When a patient is undiagnosed and their COPD stage changes	Binomial distribution with mean as probability of being diagnosed. Patients who had a severe exacerbation in the year are considered diagnosed automatically.	p(Diagnosed | COPD Severity)* [1-p(Diagnosed | COPD Severity)]
Drug therapy	If a patient is diagnosed, identify which type of drug therapy they are taking	At model entry and at change in COPD severity level	Discrete random variable Possible drugs are ICS, LAMA, LABA, ICS-LABA, LAMA-LABA, MITT, SITT, or None. Distribution based on observed distributions in claims data.	*p*(Drug Treatment | Previous Drug Treatment, COPD Severity, and Payer Type)
Adherence	Patient's proportion of days covered, if they are taking a drug therapy	Annually as long as a patient is on a drug therapy	Continuous uniform between 25 (minimum adherence) and 80 (full adherence)	1/(80 - 25)
No. of severe exacerbations	Patient's number of severe exacerbations over 1 year	Annually	Poisson random variable with mean based on observed rates in claims data. Adjustments to mean made on drug therapy and adherence.	Poisson [(Mean Severe Exacerbations | COPD Severity) * Impact of Triple Therapy * Impact of Adherence]
No. of moderate exacerbations	Patient's number of moderate exacerbations over 1 year	Annually	Poisson random variable with mean based on observed rates in claims data. Adjustments to mean made on drug therapy and adherence.	Poisson [(Mean Moderate Exacerbations | COPD Severity) * Impact of Triple Therapy * Impact of Adherence]

Variable	Description	Frequency	Distribution	Formula *f(x)**
Change in smoking status	Patient's smoking status for year	Annually	Discrete random variable. Probability varies based on patient's current smoking status. Eligible smoking statuses are Never, Current, or Former	*p*(Smoking Status | Previous Smoking Status)
FEV_1 decay	FEV_1 decay rate for patients	Annually	Normally distributed with mean and standard deviation based on COPD severity and smoking status. If FEV_1 decay is positive, set decay to 0. Additional decay added if the patient had exacerbation in year based on stage	Normal(mean | COPD Severity and Smoking Status, standard deviation | COPD Severity and Smoking Status) + p(1 or more exacerbation)*(decay due to exacerbation | COPD Severity)
Mortality	Did the patient die in the year?	Annually	Binomial distribution with mean as probability of dying subject to age and sex. If the patient does not die due to their age/sex mortality, Patients may also die if they had a severe exacerbation in the year.	Age Sex Death: *p*(death) * (1-*p*(death))<br><br>Exacerbation Death (if no Age/Sex Death): *p*(Death from Severe Exacerbation)) ^ No. of Severe Exacerbations

Abbreviations: Abbreviations: COPD, chronic obstructive pulmonary disease; FEV_1, forced exhalation volume in 1 second; ICS, inhaled corticosteroid; LABA, long-acting beta agonist; LAMA, long-acting muscarinic antagonist; MITT, multiple-inhaler triple therapy; SITT, single-inhaler triple therapy.

**p(x)* = probability of *x*, eg, *p*(death) = annual probability a patient dies; *p(x | y*) = probability of *x* given *y*, eg, *p*(Drug Treatment | COPD Severity) = annual probability of drug a treatment given a patient's COPD severity.
